# Glitch-free X-ray absorption spectrum under high pressure obtained using nano-polycrystalline diamond anvils

**DOI:** 10.1107/S0909049512026088

**Published:** 2012-07-11

**Authors:** Naoki Ishimatsu, Ken Matsumoto, Hiroshi Maruyama, Naomi Kawamura, Masaichiro Mizumaki, Hitoshi Sumiya, Tetsuo Irifune

**Affiliations:** a Department of Physics, Graduate School of Science, Hiroshima University, 1-3-1 Kagamiyama, Higashihiroshima, Hiroshima 739-8526, Japan; b Japan Synchrotron Radiation Research Institute (JASRI), SPring-8, 1-1-1 Kouto, Sayo-cho, Sayo-gun, Hyogo 679-5198, Japan; c Electronics and Materials R&D Laboratories, Sumitomo Electric Industries, 1-1-1 Koyakita, Itami, Hyogo 664-0016, Japan; d Geodynamics Research Center, Ehime University, 2-5 Bunkyo-cho, Matsuyama 790-8577, Japan

**Keywords:** X-ray spectroscopy, high pressure, glitch, nano-polycrystalline diamond, diamond anvil cell

## Abstract

Nano-polycrystalline diamond (NPD) [Irifune *et al.* (2003 ▶), *Nature (London)*, **421**, 599] has been used to obtain a glitch-free X-ray absorption spectrum under high pressure. In the case of conventional single-crystal diamond (SCD) anvils, glitches owing to Bragg diffraction from the anvils are superimposed on X-ray absorption spectra. The glitch has long been a serious problem for high-pressure research activities using X-ray spectroscopy because of the difficulties of its complete removal. It is demonstrated that NPD is one of the best candidate materials to overcome this problem. Here a glitch-free absorption spectrum using the NPD anvils over a wide energy range is shown. The advantage and capability of NPD anvils is discussed by a comparison of the glitch map with that of SCD anvils.

## Abstract 2

Nano-polycrystalline diamond has been used to obtain a glitch-free X-ray absorption spectrum under high pressure. The advantage and capability of nano-polycrystalline diamond anvils is discussed by a comparison of the glitch map with that of single-crystal diamond anvils.

## 1. Introduction

Diamond is an ideal material for the anvils used in high-pressure research activities using synchrotron radiation because of its hardness and low absorbance in the hard X-ray region. A diamond anvil cell (DAC) has recently been utilized for not only X-ray diffraction but also X-ray spectroscopy (Haskel *et al.*, 2007 ▶; Kawamura *et al.*, 2009 ▶; Aquilanti *et al.*, 2009 ▶): X-ray absorption near-edge structure (XANES) and extended X-ray absorption fine structure (EXAFS), and X-ray magnetic circular dichroism (XMCD). These spectroscopic techniques enable the investigation of pressure-induced changes in the local atomic structure, electronic structure and magnetic states of both the crystalline and the non-crystalline materials. After the development of perforated diamond anvils in order to reduce the X-ray absorption itself (Dadashev *et al.*, 2001 ▶), the coverage of X-ray absorption experiments under high pressure extends to lower energies; high-pressure research at the Ce *L*_3_-edge (5.723 keV) (Itié *et al.*, 2005 ▶; Rueff *et al.*, 2011 ▶) and Ti *K*-edge (4.966 keV) (Itié *et al.*, 2006 ▶; Jaouen *et al.*, 2007 ▶) have been reported.

The most serious problem associated with X-ray spectroscopy under high pressure is Bragg diffraction from the diamond anvils. Because a conventional anvil is made of a single-crystal diamond (SCD), when the Bragg law is satisfied in the diamond at a photon energy *E* the transmitted intensity is dramatically reduced by the Bragg diffraction, and a spike-like peak called a ‘glitch’ is superimposed on the original spectrum of the sample at *E*. This glitch causes severe distortion of the spectrum; therefore, considerable efforts have been expended to prevent Bragg diffraction (Sapelkin & Bayliss, 2001 ▶; Itié *et al.*, 2005 ▶; Hong *et al.*, 2009 ▶; Bhalerao *et al.*, 2010 ▶). Because the Bragg condition is sensitive to the orientation of the diamond anvil with respect to the incident X-ray, we can effectively reduce the number of glitches by rotating the DAC to a suitable angle. In a recent paper, Hong *et al.* (2009 ▶) reported that glitches were removed from the EXAFS spectra at the Ge *K*-edge using an iterative algorithm based on repeated measurements over a small angular range of DAC orientation, *e.g.* within ±3° relative to the direction of the X-ray beam. However, this procedure is time consuming and may be difficult in those energy regions where Bragg diffraction is frequently encountered. To the best of our knowledge, the complete removal of the diamond glitches is still a challenge.

**Figure 1:**
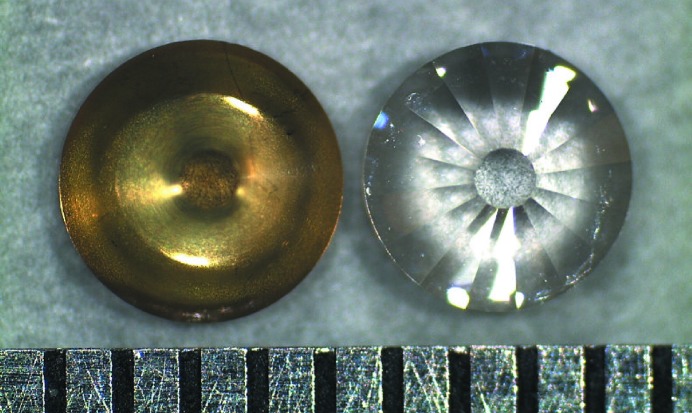
Photograph of the NPD (left) and the SCD (right) anvils used in this study. Both anvils have an outer diameter of 2.0 mm and a culet size of 0.45 mm. The height of the anvils is 1.0 mm. The scale is 0.5 mm per division. This figure will appear in color in the online version of the paper.

Nano-polycrystalline diamond (NPD) is one of the best candidate materials for eliminating glitches. High-purity single-phase NPD without any binder material has been synthesized from graphite by a direct conversion sintering method under high pressure and high temperature (Irifune *et al.*, 2003 ▶; Sumiya *et al.*, 2004 ▶). Because an NPD consists of randomly oriented diamond grains that are several tens of nanometers in size, the Bragg law is always satisfied independently of the photon energy *E*. Therefore, it is expected that the Bragg diffraction from NPD anvils provides a smooth background to the absorption spectrum in contrast to the glitches from SCD anvils. This characteristic of the NPD anvils is suitable for the X-ray spectroscopic methodology under high pressure. Fig. 1 ▶ shows a photograph of SCD and NPD anvils. Although the NPD anvil has a dark yellow color owing to nitrogen inclusion, optical transparency to observe the sample room is ensured. The hardness of NPD is comparable with or even higher than that of a SCD (Sumiya & Irifune, 2007 ▶), and the maximum pressure of the NPD anvils with a culet size of more than 300 µm is 1.5–2 times higher than that of SCD anvils (Nakamoto *et al.*, 2011 ▶). These characteristics of the NPD enable the measurements of glitch-free X-ray absorption spectra under megabar pressures. Two reports of EXAFS and magnetic EXAFS studies using the NPD anvils already exist (Matsumoto *et al.*, 2011 ▶; Baldini *et al.*, 2011 ▶). Both reports show that glitch-free spectra were obtained, and that the local structure around the absorbing atom was successfully determined.

This paper addresses the issue of glitches from a technical viewpoint. We have conducted a comparative study of the X-ray absorption spectrum obtained using the SCD and the NPD anvils. The influence of Bragg diffraction from the two anvils is discussed by a comparison of the glitch map of NPD anvils with that of SCD anvils. It is concluded that the use of the NPD anvils is highly advantageous to X-ray spectroscopy under high pressure.

## 2. Glitches from the SCD anvils

**Figure 2:**
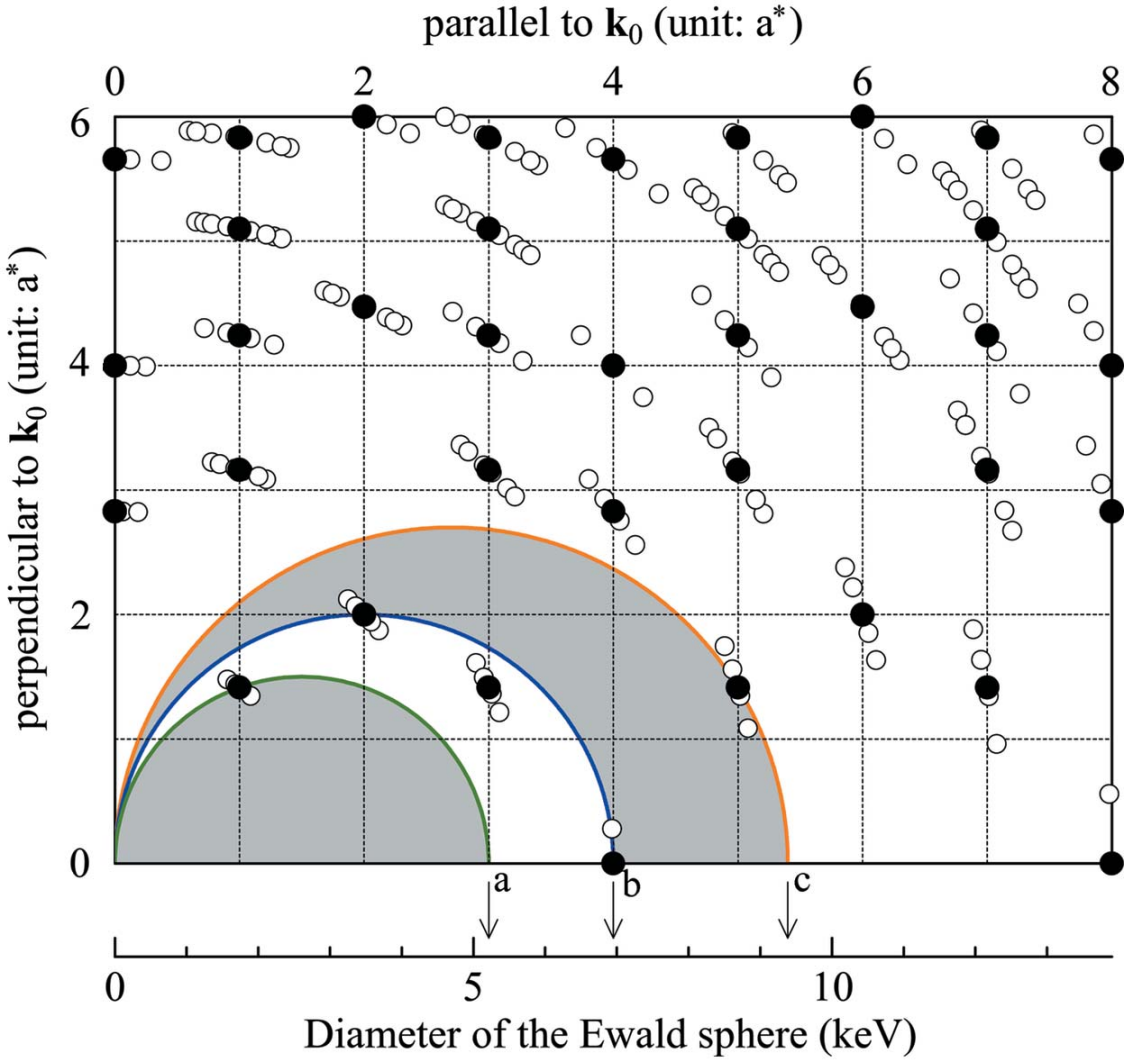
Glitch map of an SCD. The closed circles represent the reciprocal lattice points of the SCD when θ_off_ = 0°. θ_off_ corresponds to the angle between the incident X-ray **k**_0_ and the *a*-axis. The open circles are the reciprocal lattice points when θ_off_ = 4°. The lower abscissa shows the diameter of the Ewald sphere in units of the photon energy *E*. The half circles *a*, *b* and *c* correspond to Ewald spheres of *E* = 5.21 keV, 6.95 keV and 9.38 keV, respectively. The two gray regions surrounded by the Ewald spheres indicate the traces of the Ewald sphere in the energy regions where glitches appear less frequently in the case of θ_off_ = 0°. The lattice constant of the diamond is set to 3.5671 Å. This figure will appear in color in the online version of the paper.

Fig. 2 ▶ shows a glitch map of an SCD represented by the Ewald sphere in reciprocal space. In order to project all the reciprocal lattice points on the two-dimensional map, each point is plotted using the components parallel and perpendicular to the incident X-ray (wavevector **k**_0_). The diameter of the Ewald sphere increases in proportion to the photon energy. When the Ewald sphere of a particular value of *E* touches an *hkl* lattice point, an *hkl* reflection occurs and a glitch appears at that *E*. Because commercially available diamond anvils are symmetrically cut around their *a*-axis with several degrees of offset angle (Sapelkin & Bayliss, 2001 ▶), two cases are considered in the glitch map: θ_off_ = 0° and θ_off_ = 4°, where θ_off_ represents the angle between **k**_0_ and the *a*-axis. In this study, θ_off_ of six SCD anvils was determined by the Laue diffraction patterns, and the average θ_off_ with its standard deviation was estimated to be 4° ± 2°. The glitch map of θ_off_ = 4° corresponds to a geometry where the SCD anvil is rotated by 1.8° around the horizontal axis and 3.6° around the vertical axis. We suppose that both rotation axes are perpendicular to **k**_0_, and each axis is parallel to the *b*- or *c*-axis of the SCD anvil when θ_off_ = 0°. The crystallographically equivalent reciprocal lattice points are located at the same position for θ_off_ = 0°, whereas they are concentrically scattered around the original position for θ_off_ = 4°. In the case of θ_off_ = 0°, there are two energy ranges where the reciprocal lattice point is absent: *E* ≤ 5.21 keV and 6.95 keV ≤ *E* ≤ 9.38 keV. In the energy range above *E* = 9.38 keV, the reciprocal lattice points are densely distributed, therefore the removal of the glitches is more difficult. The absorption experiment in the energy range *E* ≤ 5.21 keV is quite difficult because of the large absorption of X-rays (Dadashev *et al.*, 2001 ▶). Hence, the energy range for a glitch-free spectrum of the SCD anvils is practically limited within the narrow range 6.95 keV ≤ *E* ≤ 9.38 keV. Furthermore, the scattered reciprocal lattice points effectively increase the number of glitches in the case of θ_off_ = 4°. As θ_off_ increases, glitches appear more frequently at different values of *E*, and consequently the glitch-free region becomes narrower.

It should be noted that the frequency of the glitches is also related to (i) the distortion of the anvil and (ii) the relative orientation of the upper and lower anvils. At higher pressure the strain gradient is significantly introduced in the diamond anvil, which can increase the mosaic spread and broaden the glitches (Hong *et al.*, 2009 ▶). The difference between θ_off_ of the upper and lower anvils multiplies the number of glitches; therefore, it is more feasible to mount two anvils such that their *a*-axes align in the same direction. Thus it seems that the complete removal of a glitch is hardly realized when the SCD anvils are utilized.

## 3. X-ray absorption spectra using the SCD and NPD anvils

**Figure 3:**
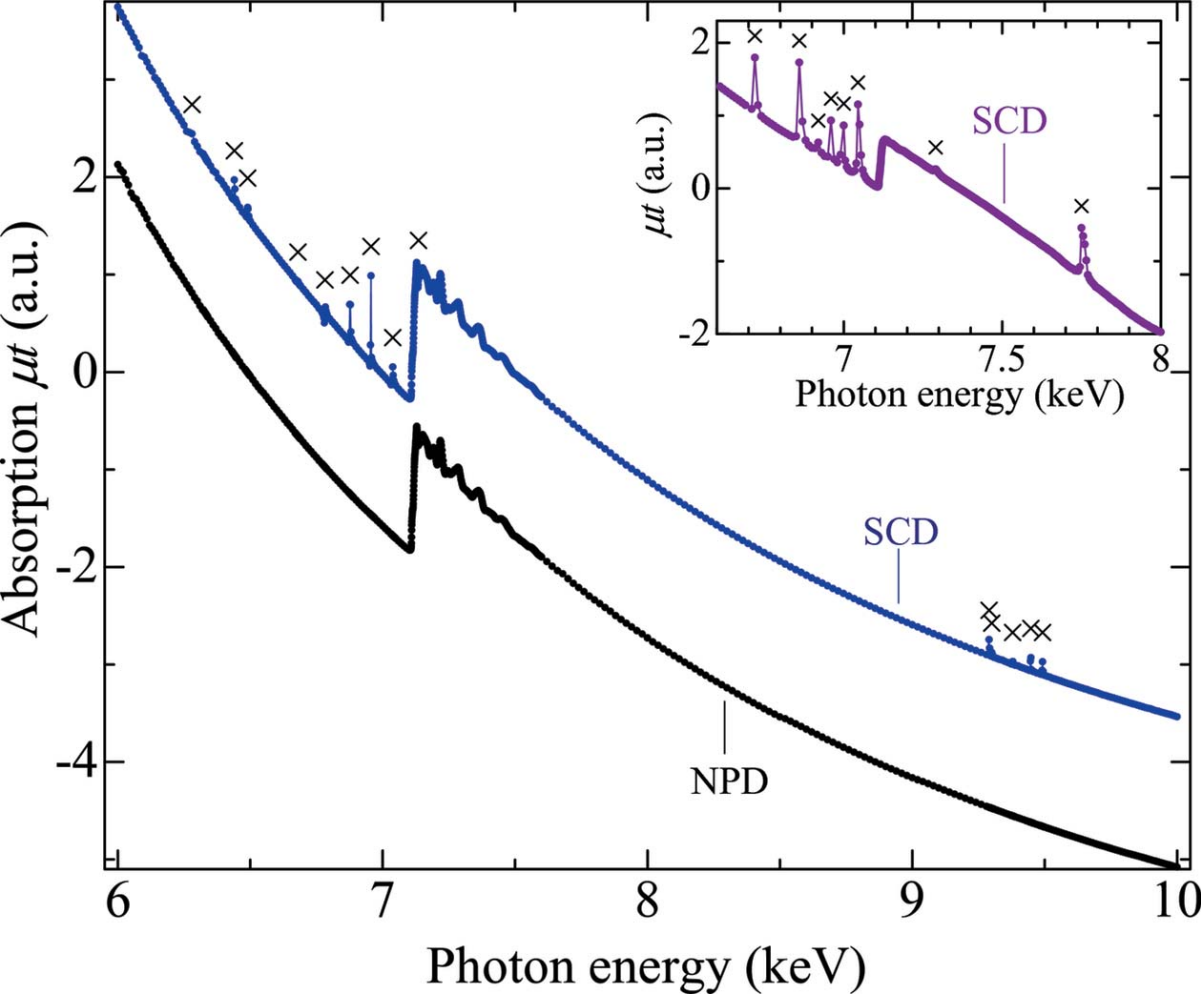
X-ray absorption spectra of an Fe foil in a DAC using the SCD and NPD anvils. Crosses indicate the glitches due to Bragg diffraction from the SCD anvils. For clarity the spectrum using the NPD anvils is shifted downward. The inset shows an X-ray absorption spectrum of an Fe-based compound around the Fe *K*-edge using a different pair of SCD anvils. Glitches observed at both baseline and EXAFS region are caused by a large θ_off_. This figure will appear in color in the online version of the paper.

Fig. 3 ▶ shows the X-ray absorption spectra of an Fe foil using the SCD or NPD anvils over a wide energy range from 6 keV to 10 keV. The spectra were measured on BL39XU of the SPring-8 synchrotron radiation facility (Kawamura *et al.*, 2009 ▶). We measured these spectra by sandwiching the Fe foil, 5 µm in thickness, with two anvils without pressure. The thickness of each anvil is about 1 mm. The incident and transmitted beams were measured using ionization chambers. Higher harmonics of the incident beam were reduced by a Rh-coated mirror, and the incident beam was focused at the sample position by Kirkpatrick–Baez mirrors (Yumoto *et al.*, 2009 ▶). The spectra were obtained by a conventional scanning method.

The spectrum of the SCD anvils shows many glitches at energy values around *E* = 6.4, 6.9 and 9.4 keV. The sharp and strong Bragg diffraction from the SCD anvils deforms mainly the baseline of the absorption profile. According to the glitch map, the glitches around 6.4 keV, 6.9 keV and 9.4 keV are assigned to 311, 220 + 400 and 511 reflections, respectively. The low density of glitches in the range from 7.2 to 9.2 keV is fairly consistent with the glitch-free region. The distribution of the glitches indicates that θ_off_ of the anvils is less than 1.5°. However, as mentioned above, the glitch-free region becomes narrow when θ_off_ increases. The inset in Fig. 3 ▶ shows an X-ray absorption spectrum of an Fe-based compound, and the spectrum was obtained using a different pair of commercially available SCD anvils. Glitches are observed not only in the baselines but also in the EXAFS spectrum. Two glitches in the EXAFS region are assigned to the 220 reflections, indicating that one of the SCD anvils possesses a large offset angle to be θ_off_ ≃ 6.0°. Compared with other reflections, the equivalent reciprocal lattice points of the 220 reflection rapidly move away from the Ewald sphere of *E* = 6.95 keV with increasing θ_off_. Consequently, the glitches from the 220 reflection are dispersed in the wide range of the absorption spectrum including the EXAFS region.

**Figure 4:**
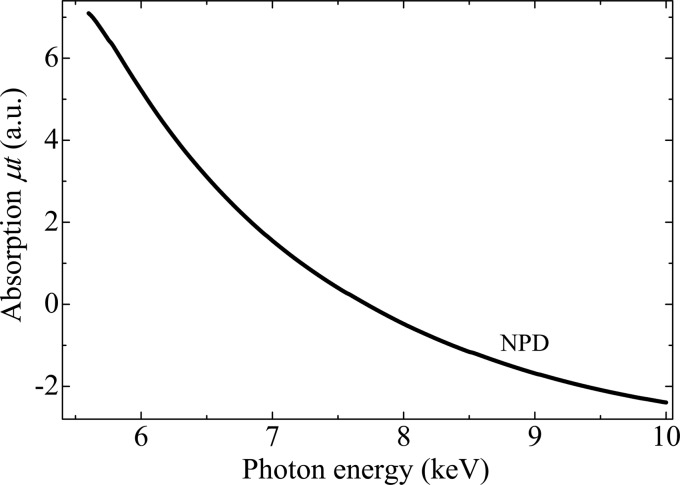
X-ray absorption spectrum of the NPD anvils. This spectrum was measured without a sample foil.

In contrast, a glitch-free spectrum is obtained by the use of the NPD anvils; Fig. 3 ▶ shows that the spike-like peaks attributed to the glitches vanish in the spectrum of NPD in the wide energy range. The absorption coefficient changes smoothly below and above the Fe *K*-absorption edge, and high-quality XANES and EXAFS spectra of the Fe foil are realized. Fig. 4 ▶ shows the absorption profile of the NPD anvils without the Fe foil over a wide energy range from 5.6 keV to 10 keV. The absorption of NPD anvils gradually increases with decreasing *E* because of the large absorption coefficient of diamond in the lower energy range (Sapelkin & Bayliss, 2001 ▶). Additional absorption edges attributed to impurities are not observed, indicating that the NPD anvil is made of high-purity single-phase diamond without any binder material. Therefore, the obtained absorption profile demonstrates that the NPD anvil is very suitable for X-ray absorption measurements under high pressure.

## 4. Influence of Bragg diffraction from NPD anvils

**Figure 5:**
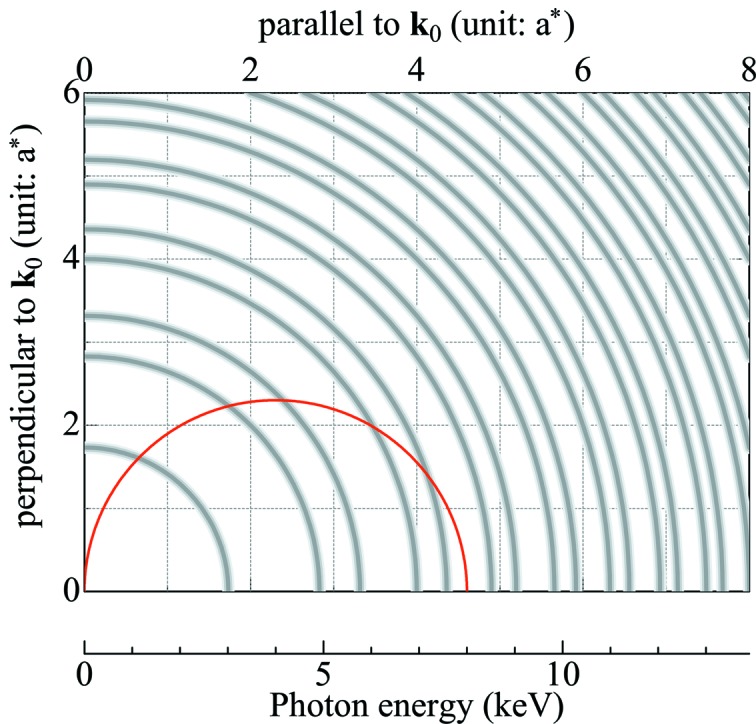
Glitch map of an NPD. Broadening lines of the quarter circles represent the class of the equivalent reciprocal lattice points owing to randomly oriented diamond grains. As an example, the Ewald sphere of *E* = 8 keV is represented by the solid line. This figure will appear in color in the online version of the paper.

As shown in the glitch map of the NPD (Fig. 5 ▶), the equivalent reciprocal lattice points of the NPD are arranged in a concentric pattern. Randomly oriented nano-diamond crystals in the NPD (Sumiya *et al.*, 2004 ▶) result in the uniform distribution of the reciprocal lattice points on the circles. This implies that the intensity of the diffracted X-ray from NPD anvils is constant or changes moderately with respect to *E*. Consequently, the NPD imparts a smooth background to the absorption profile.

**Figure 6:**
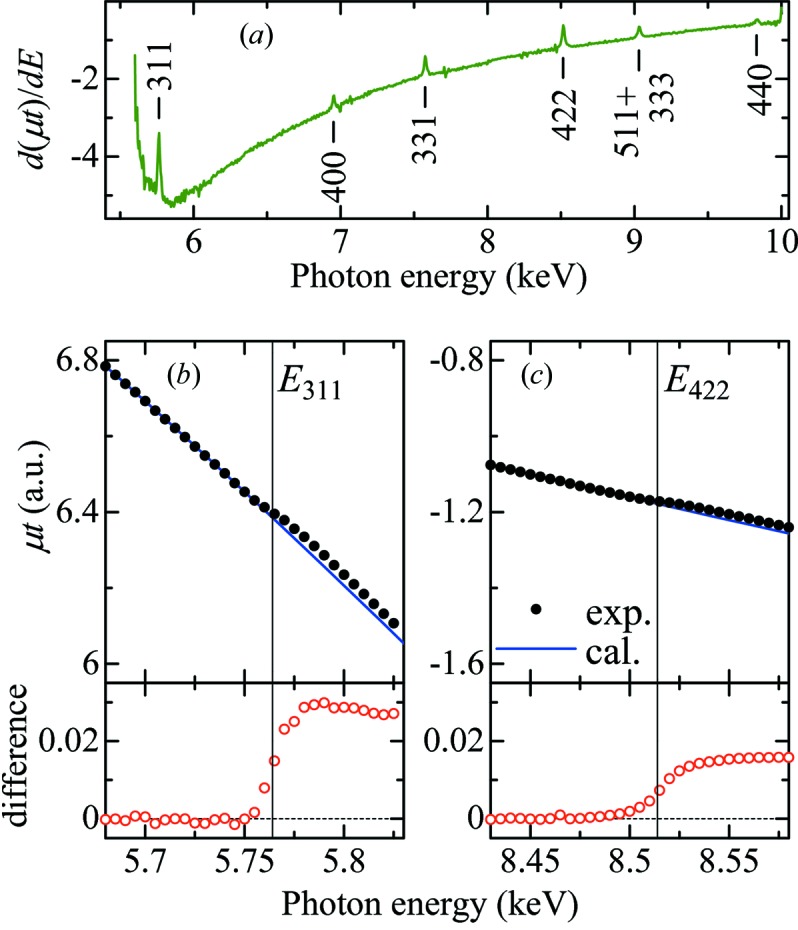
(*a*) First-derivative profile of the X-ray absorption spectrum, dμ*t*/d*E*, of the NPD anvils. Ticks near the profile indicate peaks due to *hkl* Bragg reflection from the anvils. (*b*) and (*c*) Solid circles in the upper panels denote the enlarged plots of the absorption spectrum using the NPD anvils at around (*b*) *E*_311_ = 6.952 keV and (*c*) *E*_422_ = 8.514 keV. The solid lines represent the fitted curves based on the Victoreen equation (see text). The open circles in the lower panels correspond to the difference between the experimental data and the fitted curves. The vertical lines indicate the position of *E*_*hkl*_ where *hkl* reflection occurs. This figure will appear in color in the online version of the paper.

As *E* increases, the Ewald sphere increases in size and eventually intersects with the outer circles of the higher-order reciprocal lattice points. This peculiar geometry produces new diffraction from the NPD anvil with Bragg angle θ_B_ = 90°. In the range 5.6 keV ≤ *E* ≤ 10 keV, new diffraction occurs at *E*_311_ = 5.764 keV, *E*_400_ = 6.952 keV, *E*_331_ = 7.575 keV, *E*_422_ = 8.514 keV, *E*_511+333_ = 9.030 keV and *E*_440_ = 9.830 keV, where *E*_*hkl*_ corresponds to the energy of the first intersection with the circle of the *hkl* reflection. In Fig. 6(*a*) ▶, the first-derivative X-ray absorption spectrum of the NPD anvils, dμ*t*/d*E*, is plotted to find the influence of the new diffraction at *E*_*hkl*_, where μ is the linear X-ray absorption coefficient and *t* is the sample thickness. Six peaks are observed in the dμ*t*/d*E* profile; they are exactly located at *E*_*hkl*_. Therefore, anomalies appear in the X-ray absorption spectrum at *E*_*hkl*_ as a result of Bragg diffraction from the NPD anvils.

Figs. 6(*b*) and 6(*c*) ▶ show enlarged plots of the absorption spectrum of the NPD anvils around *E*_311_ and *E*_422_, respectively. In order to extract the effects of the Bragg diffraction on the absorption spectrum, we performed a least-squares fit of the absorption profile using the Victoreen equation: *AE* ^−3^ + *BE* ^−4^ + *C*, where coefficients *A*, *B* and *C* are the fitting parameters. Each least-squares fit was made at energies below *E*_*hkl*_, and the difference between the fitted curve and the experimental data is plotted. Both figures show that the absorption profile deviates from the Victoreen equation, and a step appears at the expected energy *E*_*hkl*_. The step at *E*_311_ is largest (∼0.028) and the step at *E*_422_ is second largest (∼0.016) in the energy range of this study. The magnitude of all steps is of the order of 10^−2^ or less, which is small compared with the rapid decay of the absorption profile. Hence, the influence of these steps is almost negligible for data analysis if the sample thickness is carefully prepared to keep the edge jump Δμ*t* ≃ 1 (Bunker, 2010 ▶).

The magnitude of the step depends on the intensity of the *hkl* reflection. Therefore, below *E* = 5.6 keV the lower-order 111 and 220 reflections probably cause a large step at *E*_111_ = 3.010 keV and *E*_220_ = 4.916 keV, respectively. On the other hand, above *E* = 10 keV it is inferred that steps owing to the higher-order reflections are less effective. We emphasize that the step is easily found and removed during data analysis; because *E*_*hkl*_ is independent of the alignment of the NPD anvils, the step owing to the *hkl* reflection is observed only at that particular *E*_*hkl*_. Since the *hkl* reflection broadens with increasing strains in the NPD anvils at high pressures, it is considered that the step is influential in the data analysis only at a low-pressure region.

## 5. Concluding remarks

We have shown the difficulties in removing glitches owing to diffraction from the SCD anvils, and have presented a new application using the NPD anvils for glitch-free absorption spectra. The nano-polycrystalline structure of the NPD anvil enables us to obtain an absorption spectrum without glitches even under megabar pressures. Small anomalies remain in the absorption profile as a result of Bragg diffraction from the NPD anvils. However, the anomaly is almost negligible, and we can counteract the influence during data analysis. In the near future, NPD anvils would be used in the following spectroscopic techniques where glitches have been an inevitable problem so far:

(i) XANES. NPD anvils are used to perform measurements at absorption edges close to the *hkl* reciprocal points of the SCD anvils, *e.g.* Ce *L*_3_-edge, Eu *L*_3_-edge, Mn *K*-edge, Fe *K*-edge and the edges located above a photon energy of 9.38 keV.

(ii) EXAFS. A glitch-free EXAFS profile with a wide energy range of more than 1 keV is obtained. The NPD anvils stimulate the structural study of crystal, quasicrystal and glass materials under high pressure (Baldini *et al.*, 2011 ▶).

(iii) XMCD and magnetic EXAFS. NPD anvils prevent the modification of the circular polarization of the incident beam that is caused by the strong Bragg diffraction from the SCD anvils. This ensures high-quality dichroic spectra with a wide energy range (Matsumoto *et al.*, 2011 ▶).

(iv) Density measurement. NPD anvils can be used for density measurements by using the transmitted X-rays from a sample if the thickness of the sample is accurately evaluated (Sato & Funamori, 2008 ▶).

## References

[bb1] Aquilanti, G., Mathon, O. & Pascarelli, S. (2009). *J. Synchrotron Rad.* **16**, 699–706. 10.1107/S0909049509037571 19844002

[bb2] Baldini, M., Yang, W., Aquilanti, G., Zhang, L., Ding, Y., Pascarelli, S. & Mao, W. L. (2011). *Phys. Rev. B*, **84**, 014111.

[bb3] Bhalerao, G. M., Polian, A., Gauthier, M., Itié, J.-P., Baudelet, F., Ganguli, T., Deb, S. K., Mazher, J., Pagès, O., Firszt, F. & Paszkowicz, W. (2010). *J. Appl. Phys.* **108**, 083533.

[bb4] Bunker, G. (2010). *Introduction to XAFS.* Cambridge University Press.

[bb5] Dadashev, A., Pasternak, M. P., Rozenberg, G. K. & Taylor, R. D. (2001). *Rev. Sci. Instrum.* **72**, 2633–2637.

[bb6] Haskel, D., Tseng, Y. C., Lang, J. C. & Sinogeikin, S. (2007). *High Press. Res.* **78**, 083904. 10.1063/1.2773800 17764332

[bb7] Hong, X., Newville, M., Prakapenka, V. B., Rivers, M. L. & Sutton, S. R. (2009). *Rev. Sci. Instrum.* **80**, 073908. 10.1063/1.3186736 PMC2730721 19655966

[bb8] Irifune, T., Kurio, A., Sakamoto, S., Inoue, T. & Sumiya, H. (2003). *Nature (London)*, **421**, 599–600. 10.1038/421599b 12571587

[bb9] Itié, J. P., Baudelet, F., Congeduti, A., Couzinet, B., Farges, F. & Polian, A. (2005). *J. Phys. Condens. Matter*, **17**, S883.

[bb10] Itié, J. P., Couzinet, B., Polian, A., Flank, A. M. & Lagarde, P. (2006). *Europhys. Lett.* **74**, 706–711.

[bb11] Jaouen, N., Dhaussy, A. C., Itié, J. P., Rogalev, A., Marinel, S. & Joly, Y. (2007). *Phys. Rev. B*, **75**, 224115.

[bb12] Kawamura, N., Ishimatsu, N. & Maruyama, H. (2009). *J. Synchrotron Rad.* **16**, 730–736. 10.1107/S0909049509034700 19844006

[bb13] Matsumoto, K., Maruyama, H., Ishimatsu, N., Kawamura, N., Mizumaki, M., Irifune, T. & Sumiya, H. (2011). *J. Phys. Soc. Jpn*, **80**, 023709. 10.1107/S0909049512026088 PMC3621395 22898956

[bb14] Nakamoto, Y., Sakata, M., Sumiya, H., Shimizu, K., Irifune, T., Matsuoka, T. & Ohishi, Y. (2011). *Rev. Sci. Instrum.* **82**, 066104. 10.1063/1.3600794 21721739

[bb15] Rueff, J. P., Raymond, S., Taguchi, M., Sikora, M., Itié, J. P., Baudelet, F., Braithwaite, D., Knebel, G. & Jaccard, D. (2011). *Phys. Rev. Lett.* **106**, 186405. 10.1103/PhysRevLett.106.186405 21635111

[bb16] Sapelkin, A. V. & Bayliss, S. C. (2001). *High Press. Res.* **21**, 315–329.

[bb17] Sato, T. & Funamori, N. (2008). *Rev. Sci. Instrum.* **79**, 073906. 10.1063/1.2953093 18681715

[bb18] Sumiya, H. & Irifune, T. (2007). *J. Mater. Res.* **22**, 2345–2351.

[bb19] Sumiya, H., Irifune, T., Kurio, A., Sakamoto, S. & Inoue, T. (2004). *J. Mater. Sci.* **39**, 445–450.

[bb20] Yumoto, H., Hirata, K., Nisawa, A., Ueno, G., Sato, M., Son, J.-Y., Koganezawa, T., Machida, M., Muro, T., Hirosawa, I., Suzuki, M., Kawamura, N., Mizumaki, M., Ohashi, H., Yamamoto, M., Watanabe, Y. & Goto, S. (2009). *Proc. SPIE*, **7448**, 74480Z.

